# Hygiene Behaviors Associated with Influenza-Like Illness among Adults in Beijing, China: A Large, Population-Based Survey

**DOI:** 10.1371/journal.pone.0148448

**Published:** 2016-02-03

**Authors:** Shuangsheng Wu, Chunna Ma, Zuyao Yang, Peng Yang, Yanhui Chu, Haiyan Zhang, Hongjun Li, Weiyu Hua, Yaqing Tang, Chao Li, Quanyi Wang

**Affiliations:** 1 Institute for Infectious Disease and Endemic Disease Control, Beijing Center for Disease Prevention and Control, Beijing, China; 2 Division of Epidemiology, The Jockey Club School of Public Health and Primary Care, The Chinese University of Hong Kong, Hong Kong SAR, China; 3 Department of Epidemiology, Xicheng District Center for Disease Prevention and Control, Beijing, China; 4 Department of Epidemiology, Dongcheng District Center for Disease Prevention and Control, Beijing, China; 5 Department of Epidemiology, Tongzhou District Center for Disease Prevention and Control, Beijing, China; 6 Department of Epidemiology, Haidian District Center for Disease Prevention and Control, Beijing, China; 7 Department of Epidemiology, Changping District Center for Disease Prevention and Control, Beijing, China; 8 Department of Epidemiology, Huairou District Center for Disease Prevention and Control, Beijing, China; The University of Tokyo, JAPAN

## Abstract

The objective of this study was to identify possible hygiene behaviors associated with the incidence of ILI among adults in Beijing. In January 2011, we conducted a multi-stage sampling, cross-sectional survey of adults living in Beijing using self-administered anonymous questionnaires. The main outcome variable was self-reported ILI within the past year. Multivariate logistic regression was used to identify factors associated with self-reported ILI. A total of 13003 participants completed the questionnaires. 6068 (46.7%) of all participants reported ILI during the past year. After adjusting for demographic characteristics, the variables significantly associated with a lower likelihood of reporting ILI were regular physical exercise (OR 0.80; 95% CI 0.74–0.87), optimal hand hygiene (OR 0.87; 95% CI 0.80–0.94), face mask use when going to hospitals (OR 0.87; 95% CI 0.80–0.95), and not sharing of towels and handkerchiefs (OR 0.68; 95% CI 0.63–0.73). These results highlight that personal hygiene behaviors were potential preventive factors against the incidence of ILI among adults in Beijing, and future interventions to improve personal hygiene behaviors are needed in Beijing.

## Introduction

Respiratory pathogens such as seasonal influenza are a major cause of global morbidity and mortality each year, posing threats to the health of the population worldwide [1]. Influenza-like illness (ILI) is a medical diagnosis of possible influenza or other illness causing a set of common symptoms, and ILI data such as ILI rate and ILI count can to some extent reflect the activity of influenza and other respiratory illness [2]. Although some evidence suggests that use of vaccines or antiviral are sufficient to reduce the spread of the diseases, these interventions are virus specific, with unknown effectiveness in epidemic disease [3–4]. On the other hand, personal protective measures such as use of face masks, optimal hand hygiene, and some other physical interventions have been shown to be effective in reducing the transmission of these diseases [5–6]. In addition, they are cheap and easy to be implemented and thus recommended by official institutions [7].

To date, few large-scale population surveys have been performed to describe the prevalence of these hygiene behaviors and examine whether such behaviors are associated with these diseases in developing countries where the burden of influenza and other respiratory illness are heavy and cheap interventions are needed [5–6]. In the present study, we conducted a large population-based cross-sectional survey to examine whether selected hygiene behaviors were associated with the incidence of ILI among adults in Beijing, with a hope to provide an evidence basis for future prevention and control of influenza and other respiratory illness epidemic.

## Materials and Methods

### Participants and survey design

The target population was Chinese adults living in households in Beijing. Beijing is the capital of China with a population of nearly 20 million as of 2010, and it is divided into 16 districts, which are classified as urban and suburban districts according to the population density and local economic level. A multistage stratified sampling approach was used to recruit participants in January 2011. Considering different age groups (18–29, 30–39, 40–49, 50–59 and 60+), residence (urban or suburban), and gender (male or female), a total of 20 layers were required. We estimated a sample size of 576 participants per layer by the function n = μ_α_^2^×π×(1-π)/δ^2^×deff, based on μ_α_ = 1.96, self-reported incidence of ILI (π) = 50%, maximum permissible error (δ) = 0.1π, and the design effect of complex sampling (deff) = 1.5. Regarding 20 layers and a no-answer rate of 15%, the optimal sample size for the present study was 13,248 (576 participants per layer×20 layers×1.15).

Three urban districts and three suburban districts were randomly chosen to be sampled. Five towns or streets were randomly selected from each district, and then five communities or villages were randomly selected in each of these towns or streets. In total, 150 residents’ committees or villages were confirmed as survey locations. To meet the sample size requirement, about 88 participants needed to be selected at random from each location. To do this, all households in each location were numbered according to the address numbers. Because the family size of Beijing residents was ranged from 2 to 3, approximately 29 to 44 households of them were randomly selected for interview. The interviewers visited these households one by one, and interviewed each adult within these households until 88 residents were investigated in each location.

### Data collection

The survey was carried out using a self-administered, anonymous questionnaire, which was available in Chinese and took fifteen to twenty minutes to be completed. The questionnaire consisted of five parts: (1) demographic information (gender, age, educational level, residential district name, highest education, and occupation); (2) history of ILI: Do you have had ILI (defined as fever (temperature ≥38°C) and cough or sore throat) within the past year; (3) Knowledge regarding influenza listed as follows: ‘When were the high-occurrence seasons for the flu in Beijing? ‘, ‘What is the typical clinical symptoms of the flu? ‘, ‘Is the flu same as a cold? ‘, and ‘Which is the best way for influenza prevention? ‘; (4) Health skills listed as follows: ‘Can you understand drug instructions? ‘, ‘Can you read popular science readings about infectious diseases? ‘, ‘Can you understand the results of a routine blood test? ‘, and ‘Can you use thermometer? ‘; (5) Hygiene behaviors listed as follows: ‘How many days did you do physical exercise for more than 30 minutes per day in the past week? ‘, ‘Do you wash your hands properly with soap or hand sanitizers? ‘, ‘Do you wear face mask when going to hospitals ‘, ‘Do you often open windows and doors to keep the air circulation during the respiratory infectious diseases epidemics? ‘, ‘Do you avoid going to the crowed places during the respiratory infectious diseases epidemics? ‘, ‘Do you share towels or handkerchiefs with others? ‘, and ‘Do you have accepted influenza vaccines during the last influenza season? ‘.

The value of questions was assigned according to the following principles. One point was assigned when the respondent’s answer to a question was correct or positive, and zero point was assigned if the answer to the question was incorrect or negative. For the question about frequency of physical exercise, one point was assigned to people who answered “≥3 days”.

All investigators were well trained in interviewing skills and familiar with the content of questionnaire for this study. Most of them were local healthcare workers with a bachelor degree in epidemiology or general medicine. In order to obtain as high response rates as possible, household visits were undertaken by the investigators who had good relationships with the participants and knew how to motivate them. Prior to each household visit, the investigators made an appointment with the family. Re-visits were made to the households of which all the residents were not available for the first visit. In most cases, respondents completed the questionnaire by themselves. For the respondents who could not understand the questionnaires, the investigators would read and explain the questionnaires to them.

### Statistical analysis

The questionnaire data were entered independently by two investigators and verified using Epidata software V.3.1. All the statistical analyses were carried out using SPSS Version 13.0 (SPSS Inc, Chicago, IL). The main outcome variable was self-reported ILI within the past year. Descriptive analyses were performed to generate frequency distributions of the survey variables. Difference among the subgroups were tested by Pearson’s Chi-square test with a two-sided p value <0.05 considered to be statistically significant. Possible determinants of self-reported ILI were investigated by multivariate logistic regression. Demographic characteristics of participants, score of knowledge, score of health skills, and hygiene behaviors were included as independent variables. Adjusted odds ratios with 95% confidence intervals (95% CI) evaluated the magnitude of the association between self-reported ILI and the possible determinants.

### Ethics statement

This study was approved by the Institutional Review Board and Human Research Ethics Committee of Beijing Center for Disease Prevention and Control. At the beginning of each interview with the participants, a written consent was obtained from the participants with anonymity guaranteed.

## Results

### Demographic characteristics of participants

In total, 13003 (97.9%) of the 13287 participants that we approached completed the survey. Of them, 6715 (51.7%) were female and 6427 (49.4%) lived in urban areas. The distribution of age was as follows: 18–29: 20.7% (n = 2697), 30–39: 19.5% (n = 2542), 40–49: 20.0% (n = 2600), 50–59: 20.3% (n = 2642), more than 60 years: 19.4% (n = 2522). 1798 (13.9%) participants reported a low education level (illiterate or primary school), while the others reported a relatively higher education level. (Table 1)

**Table 1:** Demographic characteristics of participants and self-reported ILI by demographic characteristics.

Demographic characteristics	Participants		Participants reporting ILI			
	No. of subjects	%	No. of cases	%	Chi-square value^§^	P value
Residence						
Urban	6427	49.4	3013	46.9	0.234	0.628
Suburban	6576	50.6	3055	46.5		
Gender						
Male	6280	48.3	2866	45.6	5.075	0.024
Female	6715	51.7	3197	47.6		
Missing*	8	0.1	5			
Age (years)						
18~	2697	20.7	1290	47.8	2.182	0.702
30~	2542	19.5	1178	46.3		
40~	2600	20.0	1215	46.7		
50~	2642	20.3	1214	46.0		
60~	2522	19.4	1171	46.4		
Educational level						
Illiterate	386	3.0	198	51.3	37.611	<0.001
Primary school	1412	10.9	711	50.4		
Junior high school	3668	28.2	1750	47.7		
Senior high school	3688	28.4	1574	42.7		
3-year college graduate or higher	3836	29.5	1829	47.7		
Missing*	13	0.1	6			
Occupation						
Employed	5411	41.6	2541	47.0	7.895	0.162
Students	435	3.3	205	47.1		
Peasants	3810	29.3	1794	47.1		
Healthcare workers	323	2.5	168	52.0		
Retirees	2037	15.7	922	45.3		
Waiting for employment	987	7.6	438	44.4		
Overall	13003		6068	46.7		

^§^The Chi-square test was used for categorical variables.

* Missing referred to “how many people did not answer this question”.

### Report of ILI by demographic characteristics

Report of ILI by demographic characteristics is shown in Table 1. In total, 6068 (46.7%) of the 13003 participants reported ILI during the past year. The proportion of subjects with ILI was higher among female participants and those with a low education level. However, there was no significant difference across urban or suburban, age groups and the six categories of occupation.

### Report of ILI by hygiene behaviors

Of the 13003 participants, 4454 (34.3%) reported doing physical exercise regularly, 2728 (20.9%) reported using face mask when going to hospitals, and 2412 (18.5%) reported recent uptake of influenza vaccine. The other hygiene behaviors were more popular among the participants, such as optimal hand hygiene (52.5%), ventilating the room regularly (86.4%), avoidance of going to the crowded places during respiratory infectious disease epidemics (72.0%), not sharing of towels and handkerchiefs with others (66.5%). (Table 2)

**Table 2:** Self-reported ILI by hygiene behaviors.

Factors	Participants		Participants reporting ILI			
	No. of subjects	%	No. of cases	%	Chi-square value	P value
Regular physical exercise						
Yes	4454	34.3	1891	42.5	50.058	<0.001
No	7257	55.8	3568	49.2		
Unclear	1292	9.9	609	47.1		
Optimal hand hygiene						
Yes	6827	52.5	3016	44.2	36.783	<0.001
No	6154	47.3	3046	49.5		
Missing*	22	0.2				
Face mask use when going to hospitals						
Yes	2728	20.9	1154	42.5	24.179	<0.001
No	10298	79.0	4911	47.8		
Missing*	9	0.1				
Ventilating the room regularly						
Yes	11258	86.4	5222	46.5	0.866	0.352
No	1775	13.6	844	47.7		
Missing*	2	0.0				
Avoidance of going to the crowded places during respiratory infectious disease epidemics						
Yes	9390	72.0	4385	46.8	0.346	0.556
No	3643	28.0	1681	46.2		
Missing*	2	0.0				
Not sharing of towels and handkerchiefs						
Yes	8669	66.5	3725	43.1	136.2	<0.001
No	4346	33.3	2335	53.9		
Missing*	20	0.2				
Recent uptake of influenza vaccine						
Yes	2412	18.5	1214	50.3	16.23	<0.001
No	10562	81.2	4837	45.8		
Missing*	29	0.2				
Score of knowledge						
0	159	1.2	84	52.8	15.483	<0.001
1	1016	7.8	525	51.9		
2	2949	22.6	1402	47.7		
3	4711	36.2	2144	45.6		
4	3603	27.6	1630	45.4		
Missing*	591	4.5				
Score of health skills						
0	1388	10.7	681	49.1	20.548	<0.001
1	2994	23.0	1462	48.8		
2	2940	22.6	1401	47.7		
3	2748	21.1	1245	45.3		
4	2899	22.3	1269	43.8		
Missing*	34	0.3				

* Missing referred to “how many people did not answer this question”.

Report of ILI by hygiene behaviors is shown in Table 2. Chi-square tests showed that regular physical exercise, optimal hand hygiene, face mask use when going to hospitals, not sharing of towels and handkerchiefs, not receiving influenza vaccination recently, higher level of knowledge, and higher level of health skills were factors associated with a lower proportion of subjects reporting ILI.

### Multiple logistic regression analysis for factors associated with self-reported ILI

The results of multivariate analysis are shown in Table 3. After adjusting for demographic characteristics, the variables that were significantly associated with a lower likelihood of reporting ILI were regular physical exercise (OR 0.80; 95% CI 0.74–0.87), optimal hand hygiene (OR 0.87; 95% CI 0.80–0.94), face mask use when going to hospitals (OR 0.87; 95% CI 0.80–0.95), and not sharing of towels and handkerchiefs (OR 0.68; 95% CI 0.63–0.73). However, recent uptake of influenza vaccine (OR 1.24; 95% CI 1.13–1.36) was significantly associated with a higher likelihood of reporting ILI. Ventilating the room regularly, avoidance of going to the crowded places during respiratory infectious disease epidemics, score of knowledge, and score of health skills were not significantly associated with report of ILI.

**Table 3:** Multiple logistic regression analysis for factors associated with self-reported ILI.

Factors	OR	95% C.I. for OR		P value
Regular physical exercise				
Yes	0.80	0.74	0.87	<0.001
No	1.00			
Optimal hand hygiene				
Yes	0.87	0.80	0.94	<0.001
No	1.00			
Face mask use when going to hospitals				
Yes	0.87	0.80	0.95	0.003
No	1.00			
Not sharing of towels and handkerchiefs				
Yes	0.68	0.63	0.73	<0.001
No	1.00			
Recent uptake of influenza vaccine				
Yes	1.24	1.13	1.36	<0.001
No	1.00			

## Discussion

In this cross-sectional study we found that optimal hand hygiene and face mask use were significantly associated with a lower likelihood of reporting ILI. Our results are consistent with a previous randomized trial among young adults during the 2006–2007 influenza season, which found that both face mask use and hand hygiene were associated with a significant reduction of the rate of seasonal ILI [8]. A previous meta-analysis has also confirmed the effectiveness of these interventions to reduce the spread of respiratory viruses [5–6]. Another systematic review reported that the effectiveness of hand hygiene interventions varied with setting and compliance, and a significantly more reduction of respiratory infection was observed among people in low-middle-income setting [9]. These data reveal that cheap interventions targeted at improvement of personal hygiene behaviors are very important in low- and middle-income countries, such as China.

Many studies supported the idea that regular physical exercise is an effective measure for chronic disease prevention. In this study, regular physical exercise was also found to be a protective factor against the incidence of ILI. A recent meta-analysis of four studies obtained similar results that regular, moderate-intensity exercise may have an effect on the prevention of the common cold, and the risk of the common cold in exercise group was 27% lower as compared to the control group [10]. The protective effects of regular physical exercise on these infectious diseases may be partially explained by enhanced activity of several immune parameters that could be important in limiting or clearing viral infection [11]. In addition, numerous studies have revealed that behavioral interventions, particularly those focused on exercise, can enhance antibody responses to vaccination [12–13]. This suggests that exercise is an appealing behavioral intervention for infectious disease prevention.

Our results indicate that not sharing of towels and handkerchiefs can reduce the incidence of ILI by 32 percent. Towels and handkerchiefs are life's necessities for Chinese people, and nearly one-third of the respondents reported “share towels or handkerchiefs with others” in this study. Indeed, sharing towels and handkerchiefs is a risk factor of influenza and many other respiratory infectious diseases, and their pathogens can be spread from contaminated surfaces when towels and handkerchiefs are used to dry or clean face and hands. In a study from China, sharing towels was found to be a risk factor for outbreaks of respiratory infectious diseases [14]. We therefore should include “do not share towels or handkerchiefs” as a measure for these diseases prevention.

Vaccination is an effective measure to reduce influenza infection and influenza-related morbidity and mortality [15–17]. However, we observed that influenza vaccination was significantly associated with increased ILI in this study. Other studies have reported similar findings that the vaccine did not effectively protect against influenza viruses [8,18]. Several factors may explain this result. First, compared with those who have not had a vaccination, the respondents who seek vaccination may be more health-conscious and likely to report ILI. Second, other respiratory pathogens can also cause ILI in adults, but influenza vaccines cannot prevent these pathogens. During the period of this study, in addition to the influenza viruses (positive rate = 28.6%), respiratory syncytial virus (17.7%), and adenovirus (17.3%) were also found to be the main viruses responsible for acute respiratory infections [19]. Other factors such as methodology, group of population, degree of exposure, and diagnostic criteria have effect on the evaluation of influenza vaccine efficacy [8,18].

Seasonal influenza and other respiratory illness can be transmitted through the airborne route by coughing and sneezing, and to a less extent, singing and talking. Natural ventilation created by opening windows and doors provided high rates of air exchange and theoretical protection against airborne infectious agents [20]. Although potential preventive factors such as ventilating the room regularly and avoidance of going to the crowded places were included in our study, we did not observe any decreased individual risk of ILI. In contrast, a review concluded that there was sufficient evidence to demonstrate the association between ventilation in building and airborne transmission of infectious agents such as measles, tuberculosis, chickenpox, influenza, smallpox and SARS [21]. This inconsistent result may be explained by the different study sites. Most of these original studies were conducted in hospitals, while this study was conducted at homes or other living environments. Because there are more patients who coughed and sneezed in hospitals compared to other sites, people visiting hospitals are more likely to be infected by influenza and other respiratory illness via the airborne route. In contrast, there are fewer patients in other sites, and coughing and sneezing are relatively rare events during daily life [22]. Therefore, the effect of above mentioned two preventive behaviors in hospitals may be greater than in other sites. In addition, the effect of above mentioned two preventive behaviors are possibly underestimated, since people who adopted these two hygiene behaviors are more health-conscious and likely to report ILI.

This study has several limitations. First, to complete the self-administered questionnaire, the respondents had to recall their experience, which may have introduced recall bias in data collection. Second, ILI was used to represent infectious diseases, but it is possible that the symptom was caused by non-infectious diseases in some cases. Nevertheless, a study in Beijing showed that about 60% of ILI patients were caused by respiratory viruses during this study period [19]. Thus, ILI data can to some extent reflect the activity of influenza and other respiratory illness. Third, this study was not conducted during a specific emergent infectious disease pandemic. Therefore, our observations might not be generalized well to the prevention of emergent infectious diseases of which the transmission characteristics may be different from seasonal influenza and other respiratory illness.

## Conclusions

In conclusion, personal hygiene behaviors, such as hand washing, wearing face mask, regular physical exercise, and not sharing of towels and handkerchiefs were found to be associated with lower incidence of ILI among adults in Beijing. These data reveal that simple hygiene measures seem to be effective at reducing the transmission of influenza and other respiratory illness. Despite the importance of such low cost and effective measures, maintaining hygiene behaviors for long periods would be difficult. Thus, future intervention studies targeted at improvement of personal hygiene behaviors are needed in Beijing.

## Supporting Information

S1 File Data set of this study. (XLS)
